# A fast adaptive spatio-temporal fusion method to enhanced Fit-FC

**DOI:** 10.1371/journal.pone.0301077

**Published:** 2024-07-31

**Authors:** YueSheng Jiang, Kun Yang, ChunXue Shang, Yi Luo

**Affiliations:** 1 Faculty of Geography, Yunnan Normal University, Yunnan, China; 2 School of Geography, Yunnan Normal University, Yunnan, China; 3 GIS Technology Research Center of Resource and Environment in Western China, Yunnan Normal University, Yunnan, China; 4 Dean’s Office, Yunnan Normal University, Yunnan, China; Universidade Federal de Uberlandia, BRAZIL

## Abstract

Space-time fusion is an economical and efficient way to solve "space-time contradiction". Among all kinds of space-time fusion methods, Fit-FC space-time fusion method based on weight Function is widely used. However, this method is based on the linear model to depict the phase change, but the phase change in the real scene is complicated, and the linear model is difficult to accurately capture the phase change, resulting in the spectral distortion of the fusion image. In addition, pixel-by-pixel scanning with moving Windows leads to inefficiency issues, limiting its use in large-scale and long-term tasks. To overcome these limitations, this paper developed a simple and fast adaptive remote sensing image Spatio-Temporal fusion method based on Fit-FC, called Adapt Lasso-Fit-FC (AL-FF). Firstly, the sparse characteristics of time phase change between images are explored, and a time phase change estimation model based on sparse regression is constructed, which overcomes the fuzzy problem of fusion image caused by the failure of linear regression to capture complex nonlinear time phase transition in the weighted Function method, making the algorithm better at capturing details. Secondly, an adaptive window selection Function is established to overcome the problem of manually setting parameters on different data sets, improve the convenience of the algorithm and robustness of the application on different data sets, and make the algorithm simpler and more efficient. Finally, the improved AL-FF algorithm is compared with other algorithms to verify the performance improvement. Compared with the current advanced Spatio-Temporal fusion methods, AL-FF algorithm has stronger detail capture ability and can generate more accurate fusion results. In addition, the computational efficiency is significantly improved, and the efficiency is increased by more than 20 times compared with the current mainstream method.

## Introduction

Remote sensing images with high spatial and temporal resolution are of great significance for applied research in leaf area index (LAI) monitoring [1,2], forest monitoring, crop monitoring [3,4], land surface temperature (LST) monitoring [5], and gross primary productivity (GPP) monitoring [6]. Current satellite platforms are mainly categorized into two types: geostationary orbit satellites, which have high spatial resolution but low temporal resolution, and polar orbit satellites, which have higher temporal resolution but lower spatial resolution. However, both types of satellites have the spatial and temporal contradiction problem, which makes it difficult to directly obtain high spatial and temporal resolution data [7–10]. At the same time, optical remote sensing images are easily affected by clouds and other atmospheric conditions, which reduces data availability and further restricts the ability to obtain high spatial resolution images continuously. An effective way to solve this problem is to fuse high-temporal low-resolution data with high-resolution low-temporal data to get remote sensing images with high spatial and temporal resolution [11]. Spatio-temporal fusion methods have been widely used for synthetic image generation of coarse temporal resolution but acceptable spatial resolution Landsat (Landsat) and satisfactory temporal resolution but coarse spatial resolution Moderate Resolution Imaging Spectroradiometer (MODIS) or Moderate Resolution Imaging Spectroradiometer (MERIS) imagery aiming at realizing high temporal and spatial resolution image data [12,13]. In the past decade, a variety of innovative spatio-temporal data fusion methods have emerged, which are categorized into five main groups: decomposition-based methods [14–16], weight Function-based methods [17–19], Bayesian-based methods [17,20,21], learning-based methods [22–24], and hybrid-based methods [25–28].

After spatial unmixing based methods obtain classification maps from high spatial resolution images, the classification information is used for unmixing of low-resolution images in predicted time [29]. Multi-sensor multiresolution techniques were the first to use unmixing for spatio-temporal fusion and were improved by introducing a regularization term in the loss function of spectral unmixing to ensure similarity between extracted and predicted end-elements [30]. However, these methods usually assume that the class proportions of pixels in low spatial resolution imagery remain constant, making it difficult to handle spectral variations between feature classes. Among the learning-based methods, Huang and Song first proposed a sparse representation-based spatiotemporal reflectance fusion model (SPSTFM) [31], which utilizes two pairs of Landsat and MODIS image pairs, as well as MODIS imagery at the intermediate moments to predict the fine spatial resolution images at the target moment. Later, Song and Huang further proposed a spatio-temporal fusion model based on a single image pair [32], which is implemented in two stages: firstly, the spatial resolution of the pre- and post-MODIS data is improved by sparse representations; and later, the observed Landsat data are fused with the enhanced MODIS data using high-pass modulation. Recently, some spatial and temporal fusion methods based on deep learning have also been proposed. Song et al [33] used a convolutional neural network to firstly learn the nonlinear mapping relationship between the downscaled spatial resolution Landsat and MODIS images, and then learn the mapping relationship between the downscaled spatial resolution Landsat and the original Landsat images, and finally based on the high-pass modulation and weighting strategy to Zhang et al [34] proposed a two-stream convolutional neural network (StfNet), which utilizes the structural similarity between pairs of coarse spatial resolution images and fine spatial resolution images and the rich texture information in the neighboring fine images to predict the fine target moment images. All such algorithms suffer from high complexity and high consumption of computational resources [35]. Bayesian-based methods describe spatio-temporal fusion as a maximum A posteriori problem based on a Bayesian framework [36]. Decomposition-based methods can be performed using a fine image. More precisely, it requires a fine spatial resolution thematic map, which can be derived by interpreting available fine spatial resolution data [37] or from other sources, including aerial imagery or land use databases. Spatial decomposition assumes that no land use change has occurred during the period of interest and that the class ratio of the coarse image is constant across time, thus requiring higher data quality and making it difficult to have a good application in large-scale long-term tasks [38].

The weight Function-based method is the most widely used spatiotemporal fusion method, which is based on the premise of consistent spectral variations and predicts fine pixels by shifting neighborhood information within a window. The Spatio-Temporal Adaptive Reflectance Fusion Model (STARFM) [39] is the first weight-Function-based method, which obtains the predicted value of each pixel by combining the spectral variation information of similar pixels of each pixel. However, the method may produce errors in the case of complex surface variations because it assumes that the neighboring pixels have the same variation pattern as the target pixel. To enhance the performance of STARFM, many improved versions of STARFM have appeared, among which ESTARFM (Enhanced STARFM) is the most classical [18,40].ESTARFM introduces a pixel similarity weight that can better handle complex surface change situations. However, ESTARFM has a high computational complexity and demand for computational resources [18]. In addition to the methods based on the STARFM theory, other types of weight Function-based methods have been developed, such as STI-FM, SPSTFM, Fit-FC, etc. STI-FM improves the fusion results by using a complex weight Function to consider both spatial and temporal variations. However, this method is more complex, requires a large amount of computational resources, and may be difficult to deal with some complex surface variations [41]. SPSTFM employs a band-dependent weight assignment strategy that does not depend on specific sensors. However, the disadvantage of this method is that it is more sensitive to the selection of input data, which may lead to a poor fusion effect if improperly selected, and may require a large number of parameter adjustments, which is computationally inefficient [21]; the Fit-FC method is a fusion method based on a coarse and fine image pair, which utilizes a weight Function to improve the spatial resolution of the fused image. However, this method may enhance the noise of the image and may lose some detailed information during the fusion process. [19,42]. Therefore, In order to solve the problems of computational complexity, low efficiency, and weak detail capturing ability of all the above weight Function-based spatio-temporal fusion algorithms, many researches have made efforts. In recent years, for the pixel-by-pixel fusion computation based on the weight Function approach, which leads to computational inefficiency, a flexible object-level (OL) processing strategy is proposed, and in a single pair of experiments, OL Fit-FC works best. However, the algorithm is complex, and object-oriented classification is also required before fusing images, and its detail-capturing ability is also closely related to the classification result. Secondly, since fine images need to be segmented before fusion, setting parameters in this step also affects the processing efficiency and fusion results [43]. In addition, some studies have obtained hybrid algorithms based on the combination of weight Functions and other algorithms in order to get better fusion results. For example, the flexible spatio-temporal data fusion (FSDAF) and the reliable adaptive spatio-temporal data fusion method (RASDF). [25] proposed the Flexible Spatio-Temporal Data Fusion (FSDAF) method, which combines the principles of linear solution mixing models, weight Functions, and spatial interpolation; as a result, FSDAF can recover seasonal and land cover changes, but the algorithm is complex and challenging to capture complex temporal changes [44]. In addition, RASDF introduces an adaptive local decomposition model that can retrieve substantial temporal variations before filtering and assigning residuals; however, the regional window size is determined empirically in the local solution mixing step, so it is computationally inefficient [45]. To summarize, weight Function-based spatio-temporal data fusion methods suffer from three main shortcomings: the influence of model parameters [16], the inability of linear models to capture complex nonlinear temporal phase variations [40,46], and the low computational efficiency of pixel-by-pixel scanning [43]. As a result, large-scale and long-term tasks rarely use spatiotemporal fusion techniques to fill in blank image sequences [28,47]. Therefore, large-scale and long-term studies still have difficulties in using spatio-temporal fusion techniques to fill empty image sequences [39,48–50]. Fit-FC method is a very representative algorithm based on weight Function in recent years, and has unique advantages in phenological learning and operational efficiency compared with other algorithms based on weight Function. But there are also inevitable: the influence of model parameters, the inability of linear models to capture complex nonlinear phase changes, and the low computational efficiency of per-pixel scanning.

In this study, the above shortcomings of Fit-FC method are tried to solve, and three improvement strategies are proposed: Firstly, a time change estimation model based on sparse regression is constructed by exploring the sparse characteristics of the time phase change between images, which overcomes the problem of fuzzy image caused by the failure of linear regression to capture complex nonlinear time phase change in Fit-FC method. Secondly, adaptive window selection Function and weight adaptive setting Function based on image gradient information are introduced to solve the problem that parameters need to be manually set on different data sets of Fit-FC method, which improves the convenience and robustness of the algorithm on different data sets. Finally, the weight Function constructed by spectral difference and time difference is used to compensate the residual error, which makes up for the lack of fine spatial details in the residual error directly introduced by the Fit-FC method into the coarse spatial resolution image regression model, and further improves the image quality. In this study, the above three strategies are used to improve the algorithm, which realizes the simplification of the algorithm, and further improves the computational efficiency and image detail capture ability.

**Fig 1:**
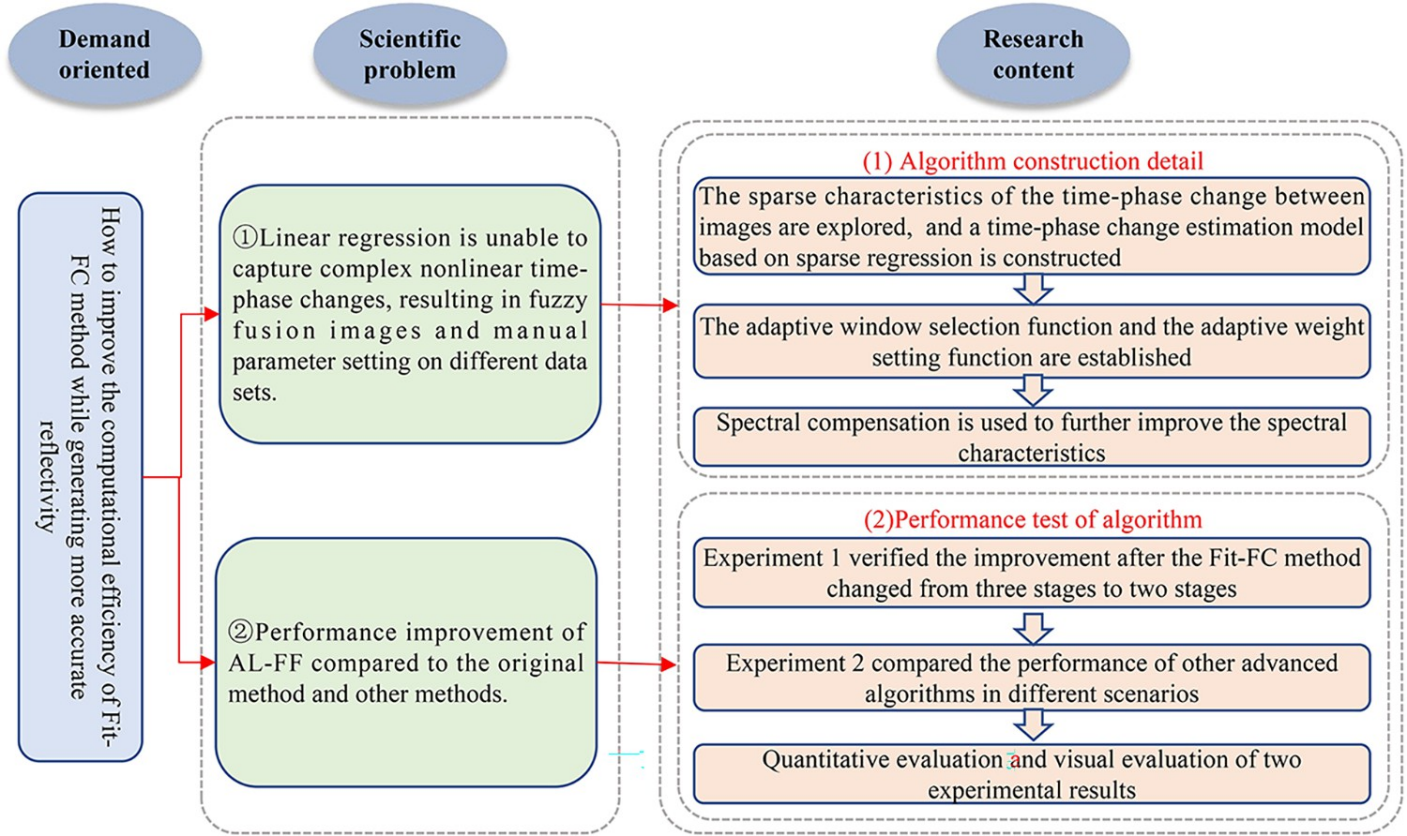
Work flow chart.

This paper is organized as follows. In Section 2, the detailed construction of the AL-FF method is presented. In Section 3, the sources of the three datasets are presented, and the experimental design of the model AL-FF is carried out: it is divided into Experiment 1 and Experiment 2. In Section 4, the AL-FF method is compared with the original algorithm at various stages, as well as experimentally with other state-of-the-art models, which are evaluated qualitatively and quantitatively. Sections 5 and 6 discuss and summarize the paper respectively. A roadmap of the work of this study is shown in Fig 1.

## AL-FF algorithm construction

Let *M*_1_, *M*_2_∈*R*^*w*×*h*×*b*^denote the coarse spatial resolution, fine temporal resolution image with *w*×*h* pixels and *B* bands for *t*_1_ and *t*_2_ time, respectively *L*_1_ and *L*_2_∈*R*^*W*×*H*×*B*^ denote the fine spatial resolution, coarse temporal resolution image with *W*×*H* pixels and *B* bands for *t*_1_ and *t*_2_ time, respectively. If there are observations *M*_1_, *M*_2_ and *L*_1_, the final fusion task is to predict the fine spatial resolution image *L*_2_ at time *t*_2_. The AL-FF method consists of two stages, adaptive sparse regression and residual compensation based on the weighting Function, respectively.

### Adaptive sparse regression (AL-RM)

In order to establish the difference in temporal variation between times *t*_1_ and *t*_2_, here we use the bootstrap filtering of localized sparse regression to establish the temporal variation relationship between coarse spatial resolution imagery at the observation moment and coarse spatial resolution imagery at the prediction moment. Specifically, first the regression model will be used to fit the relationship in a localized window of the size $\sqrt{q}\times \sqrt{q}$ of each band of the coarse spatial resolution image between the predicted and observed moments:

$$M_{2}(i,b)=\alpha (i,b)M_{1}(i,b)+\beta (i,b)+R(i,b)$$ (1)

where *α*(*i*,*b*) and *β*(*i*,*b*) are the regression coefficients for the image block with the pixel center of the *b* band of the two coarse spatial resolution images located at *i*,*R*(*i*,*b*) is the regression residual for the image block with the pixel center of the *b* band of the two coarse spatial resolution images located at *i*. Since *M*_1_ and *M*_2_ have been observed, their regression coefficients were computed by sparse regression modeling *α*(*i*,*b*):

$$\alpha (i,b)=\underset{\alpha (i,b)}{\arg\;\min}[\frac{1}{2}{\left\|{\tilde{M}}_{2}(i,b)-\alpha (i,b){\tilde{M}}_{1}(i,b)\right\|}_{2}^{2}+\lambda {\left\|\alpha (i,b)\right\|}_{1}]$$ (2)

Where ${\tilde{M}}_{1}(i,b)$ and ${\tilde{M}}_{2}(i,b)$ are *M*_1_(*i*,*b*) and *M*_2_(*i*,*b*) after de-mean, the purpose is to eliminate the effect of image brightness variation and better highlight the details of the image,which are calculated as:

${\tilde{M}}_{1}(i,b)=M_{1}(i,b)-mean(M_{1}(i,b))$ and ${\tilde{M}}_{2}(i,b)=M_{2}(i,b)-mean(M_{2}(i,b))$, *mean*(*M*_1_(*i*,*b*)) and *mean*(*M*_2_(*i*,*b*)) are the mean values of the image blocks with the band *b* pixel center located at *i* of the two phases calculated using filtering, and *λ* are the coefficients of the sparse regular term. In a local window image, on the one hand, for a region with obvious spatial heterogeneity, the features of ground objects may only appear in a few pixels; on the other hand, for a spatially homogeneous region, the features of objects in the region can be obtained only by using the features of a few pixels. Therefore, we can well assume that the regression coefficient is sparse. Therefore, we used Lasso sparse regression model to calculate the regression coefficient *α*(*i*,*b*).

For the bias term *β*(*i*,*b*), it is calculated as:

$$\beta (i,b)=mean(M_{2}(i,b))-\alpha (i,b)\cdot mean(M_{1}(i,b))$$ (3)

Using the mean value of the image to compute the bias term inspired by the bootstrap filtering allows a better consideration of the structural and textural information of the image.

Based on the coefficients obtained above, we can then predict the resulting fine spatial resolution image at time *t*_2_:

$${\tilde{L}}_{2}(i,b)=\alpha (i,b)L_{1}(i,b)+\beta (i,b)$$ (4)

Due to the resolution gap, the residuals R in the regression model cannot be directly introduced into the generated fine spatial resolution image ${\tilde{L}}_{2}$. Instead, *R* mainly retains the important time-phase difference information of the two time-phase coarse spatial resolution images, especially spectrally, and thus the generated ${\tilde{L}}_{2}$ has a large discrepancy compared to the real *L*_2_, which will be resolved in the residuals compensation.

Since the regression coefficients are computed within a localized window, the size of the localized window will significantly affect the computation of the regression coefficients, which usually need to be set manually in traditional bootstrap filtering. In addition, in the sparse regression model, the coefficients of the parameter spare regular terms will also affect the regression results. To improve the model’s robustness, we construct an adaptive window size determination Function and a regularization parameter calculation Function in our approach.

### DeterMination of adaptive window size $\sqrt{q}$

In edge and texture regions, a larger window radius is usually needed to capture more details in the image, while in the smooth areas, setting a smaller window can reduce blurring. Therefore, selecting the window size according to different types of images can effectively improve the accuracy of regression calculation. In remote sensing images, the gradient change contains the intensity and direction of the difference in the pixel value of the image. The image will show a larger gradient in the edge and texture region. On the contrary, in the smooth region of the image, the gradient of the image will be smaller, so we adaptively determine the window size by calculating the average slope of the image:

$$\sqrt{q}=[\frac{\varepsilon (w+h)}{mean(\sum_{i,j}\sqrt{{\left(\nabla M_{2}\right)}^{2}})+\varepsilon }]$$ (5)

where $mean(\sum_{i,j}\sqrt{{\left(\nabla M_{2}\right)}^{2}})$ is the average gradient of the coarse spatial resolution image *M*_2_ at the moment of prediction and *ε* is a very small positive integer to avoid a zero denominator.

### Determination of the adaptive sparse regularization parameter *λ*

A larger *λ* will impose a stronger sparse constraint for computing the regression coefficient *α*(*i*,*b*), making more elements of *α*(*i*,*b*) zero, while a smaller *λ* imposes a weaker sparse constraint. We adaptively determine the sparse regularization parameters based on the degree of image variation and the size of the adaptive window:

$$\lambda =\frac{0.1\mathrm{var}(M_{2})}{mean(\left|M_{2}-mean(M_{2})\right|)q}$$ (6)

The variance var(*M*_2_) of the image can reflect the degree of dispersion of the pixel values in the image, i.e., the degree of variation, and the mean absolute deviation $mean(\left|M_{2}-mean(M_{2})\right|)$ of the image can reflect the centrality of the distribution of the data, and by calculating the ratio of the degree of variation to the mean absolute deviation, it is possible to whereas a smaller mean absolute deviation indicates the centrality of the distribution of the data, and a larger ratio indicates a larger change in the data, whereas a smaller ratio indicates a smaller change in the data. Additionally the size of the adaptive window will be considered, with larger ratios and smaller window radii resulting in larger *λ* values. On the contrary, a smaller ratio and a larger window radius will result in a smaller *λ* value.

### Enhanced Residual Compensation (ERC)

Due to the spatial scale difference between coarse and fine spatial resolution images, the direct introduction of the residuals of the regression model for rough spatial resolution images will lead to excessive smoothing because the residuals of coarse spatial resolution images are missing fine spatial details. By constructing a weight Function to predict the value of the center pixel, we can introduce additional information on neighboring pixels and determine the contribution of neighboring pixels to the center pixel based on the weight Function, thus predicting the center pixel value more accurately and injecting fine spatial details. Based on this, the strategy of using the weight Function is considered in our proposed method to distribute the residuals in the regression model better to obtain accurate prediction results. The residuals of the coarse resolution will be interpolated to the fine spatial resolution size, after which the residuals of the fine spatial resolution of the center pixel located at position *i* for the *b* band $\overset{\wedge }{R}(i,b)$ will be represented as a linear combination of neighboring pixels:

$$\overset{\wedge }{R}(i,b)=\sum_{j=1}^{n}Wr(j,b)$$ (7)

Where *r* is the residual value of the sampled residual at the position located in the neighborhood of the neighboring pixel located near *i*, *w* is the weight of the neighboring pixel.

We used spectral differences, as well as temporal differences, to construct the weight Functions. They are based on the assumption that pixels with closer spectral values, pixels with more relative spatial distances, and pixels with more minor temporal differences between two-time images are more likely to have similar residual values, respectively.

Spectral differences are calculated as follows:

$$S\left(j,k\right)=\sqrt{{\sum_{k=1}^{b}\left[L_{1}(j,k)-L_{1}(i,k)\right]}^{2}/b}$$ (8)

Where *j*∈[1,*n*], *n* is the number of similar pixels in the window.

The spatial difference is calculated as follows:

$$D(j,k)=1+2\sqrt{{\left(x_{j}-x_{i}\right)}^{2}}/\sqrt{q}$$ (9)

The time variance is calculated as follows:

$$T(j,k)=\sum_{k=1}^{b}\left|{\overset{\wedge }{M}}_{2}(j,k)-{\overset{\wedge }{M}}_{2}(i,k)\right|/b$$ (10)

where $\overset{\wedge }{M_{2}}$ and $\overset{\wedge }{M_{1}}$ are the *M*_2_ and of the spatial size *M*_1_ of the up-sampled to fine spatial resolution image, respectively. Based on the calculated *S*, *D* and *T*, the weights can be calculated:

$$W(j,k)=(1/C(j,k))/\sum_{j=1}^{n}\sum_{k=1}^{b}C(j,k)$$ (11)

Residuals for fine resolution will also be calculated:

$$\overset{\wedge }{R}(i,b)=\sum_{j=1}^{n}W(i,b)r(j,b)$$ (12)

Best, the final predicted moment fusion image is computed as:

$$\overset{\wedge }{L_{2}}(i,b)=\overset{\wedge }{L_{2}}+\overset{\wedge }{R_{2}}(i,b)$$ (13)

### Accuracy assessment

Using the acquired real fine image as the verification image, visual reading and correlation analysis methods are used to evaluate the accuracy of the fused image from both qualitative and quantitative aspects. The visual reading method can directly analyze the similarity between the fused image and the actual image and make a preliminary judgment on the fusion accuracy of each model. The correlation analysis method mainly uses five evaluation metrics: root mean square error (RMSE), correlation coefficient CC, relative global dimensionless ERGAS, peak signal-to-noise ratio PSNR, and average absolute error AAD [19,51,52]. These metrics are used to evaluate the similarity between the fused and actual images quantitatively.

AAD is used to measure deviation. the closer the AAD is to 0, the smaller the deviation of the predicted value from the standard value.


$$AAD=\frac{1}{n}\sum_{i=1}^{n}\left|y_{i}-x_{i}\right|$$ (14)

RMSE is used to measure the difference between images and has a value ranging from 0 to 1. The smaller the RMSE, the higher the accuracy

$$RMSE=\sqrt{\frac{1}{n}}{\sum_{i=1}^{n}\left(x_{i}-y_{i}\right)}^{2}$$ (15)

CC can reflect the spectral similarity between images, the closer CC is to 1, the higher the spectral similarity is.


$$CC=\frac{\sum_{i=1}^{n}\left(y_{i}-\bar{y})(x_{i}-\bar{x}\right)}{\sqrt{\sum_{i=1}^{n}{\left(y_{i}-\bar{y}\right)}^{2}{\left(x_{i}-\bar{x}\right)}^{2}}}$$ (16)

PSNR evaluates the structural similarity between images. The closer the PSNR is to 1, the greater the structural similarity between images.


$$PSNR=10\log(\frac{MAX^{2}}{RMSE})$$ (17)

ERGAS can measure the relative error between the image classification result and the real classification result. The smaller the value of ERGAS, the smaller the error between the classification result and the real classification result, and the higher the classification accuracy.


$$ERGAS=\frac{100}{R}\sqrt[{2}]{\frac{1}{B}\sum_{K=1}^{B}\left(\frac{RMSE(x_{i},y_{i})}{n(x_{i})}\right)}$$ (18)

In Eqs (14)–(16), *n* is the total number of image pixels; *x*_*i*_ and *y*_*i*_ denote the i-th pixel of the predicted and observed images, respectively. $\tilde{y}$ and denote $\tilde{x}$ the average of the fusion result and the observed image, respectively. In Eq (17), *MAX* is the possible maximum value of the pixel. In Eq (18), *B* denotes the number of bands.

### Data and experimental design

#### Dataes

**Fig 2:**
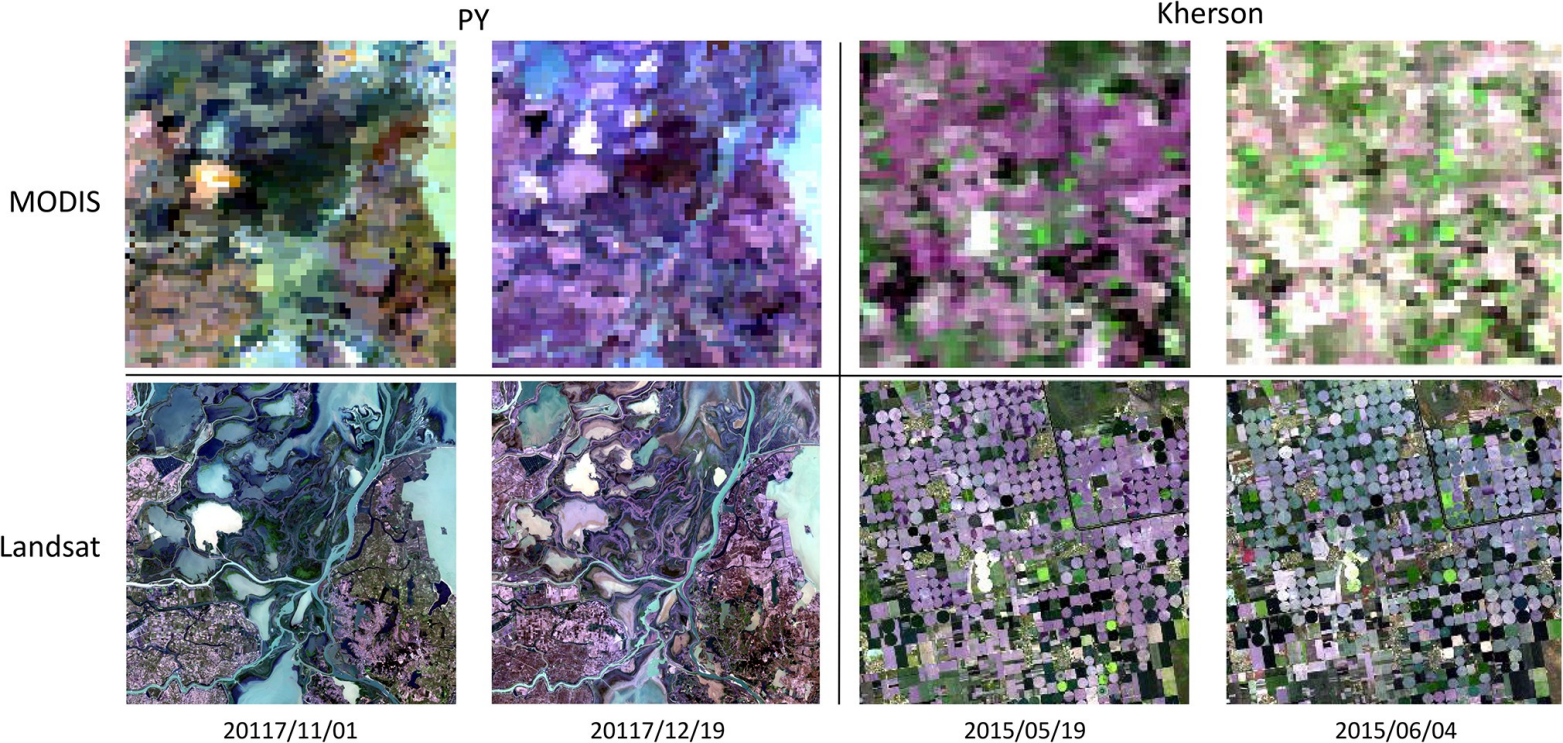
MODIS and Landsat data. Shows MODIS surface reflectance (top row) and Landsat8 surface reflectance (bottom row) images for May 19, 2015, and June 4, 2015, in the "Kherson" area, and for November 1, 2017, and December 19, 2017, in the "PY" area, respectively. Landsat8 surface reflectance (downlink) images. The "Kherson" area shows large spectral variations for different features, which are complex and heterogeneous spectral variations. In the "PY" region, it can be seen that different features have not only undergone great spatial changes but are also accompanied by significant spectral changes, thus having complex heterogeneous spatial changes and complex heterogeneous spectral changes.

We chose two real datasets for our experiments, Data 1 and Data 2, whose satellite remote sensing images are shown in Fig 2.

Data 1, two pairs of Landsat-7 ETM+ and MODIS images acquired on May 24, 2001, and July 11, 2001, respectively, located near 54°N and 104°E, a region with little spatial and seasonal variability [17]. The resolutions are 30 and 500 m, and the bands are green, red, and NIR, corresponding to bands 2, 3, and 4 of Landsat-7 ETM+ and bands 4, 1, and 2 of MODIS, respectively. Two pairs of Landsat-7 ETM+ and MODIS images acquired on May 24, 2001, were used to predict the July 11, 2001 Landsat spatial resolution images. The predicted images were compared with actual Landsat ETM+ images to evaluate the performance of the AL-FF algorithm.

(Data from: http://ledaps.nascom.nasa.gov/ledaps/Tools/StarFM.htm)

For Data 2, Landsat 8 Operational Land Imager (OLI) surface reflectance products and Moderate Resolution Imaging Spectroradiometer (MODIS) surface reflectance products were used as fine and coarse images, respectively, both in six bands, with detailed information as shown in Table 1. The preprocessing is satisfactory [43] and includes masking cloud contamination and noise regions in MODIS images based on reflectance and employing the Neighborhood-Similar Pixel Interpolator (NSPI) model [53,54] to reconstruct the information of problematic pixels. In addition, Landsat-MODIS pairs acquired on the same date were co-aligned between two images within an acceptable pixel by maximizing the correlation Function. The first study site is located in the Kherson Oblast ("Kherson") irrigation area (33°31 22.85 E, 46°40 43.55 N) in southern Ukraine, where the shape of the agricultural land is rectangular or circular, and there is a significant variation in phenology and a slight variation in shape between the selected images, but the feature types are are abundant, and different features all have large spectral variations, thus having complex heterogeneous spectral variations, and the images were acquired on May 19, 2015, and June 4, 2015, respectively. The second study site is located in the southern part of the wetland of Poyang Lake ("PY") (116°11 37.35 E, 28°57 57.39 N) in Jiangxi Province, China, which has typical southern fragmented farmland, with large and complex heterogeneous spectral variations and complex heterogeneous spatial variations. The image acquisition time is November 2017, January 1, 2017, and December 19, 2017. (Data from: https://github.com/Andy-cumt/Spatiotemporal-fusion-data).

**Table 1:** Experimental data.

Data type	Landsat 8			MODIS		
Band name	Band ID	Bandwidth (VM)	spatial resolution (m)	Band ID	Bandwidth (VM)	spatial resolution (m))
**Blue**	B2	0.45–0.51	30	B3	0.45–0.47	500
**Green**	B3	0.53–0.59	30	B4	0.54–0.56	500
**Red**	B4	0.64–0.67	30	B1	0.62–0.67	500
**NIR**	B5	0.85–0.88	30	B2	0.84–0.87	500
**SWR1**	B6	1.57–1.65	30	B6	1.62–1.65	500
**SWR2**	B7	2.11–2.29	30	B7	2.10–2.15	500

### Experimental design

The purpose of the experiments is to verify whether the improved AL-FF algorithm, based on Fit-FC, enhances the retention of structural information and significantly improves the fusion efficiency, and whether the same is true when compared with other fusion algorithms. Therefore, this study quantitatively and qualitatively compares the fusion performance of the OL method and its original method with three representative methods such as cuFSDAF, OBSTFM and RASDF. The procedures and software used for the experiments are as follows: for the OL-Fit-FC method we used the OL software processed by Guo et al [43]. We also used the eCognition software software for fine classification, and used the multi-resolution segmentation algorithm of the eCognition software to set the smoothing weights and the spectral weights to 0.5 and 0.6, respectively, with a scale value of 150.For FSDAF 2.0, RASDF, Fit-FC, and AL-FF methods were all run on the MA TLAB 2020b platform, and for RASDF, FSDAF 2.0, and Fit-FC parameters were kept the same as the default parameters in their open source codes.The parameters used in the Fit-FC fusion method were carefully selected and determined with reference to previous studies [12,19,44,55]. For Fit-FC, the RM stage contains 5×5 MODIS pixels with a resolution of 460 m in the moving window, and the SF and RC stages contain Landsat pixels with a resolution of 30 m. The number of similar pixels in Fit-FC is set to 20, and the parameter settings of the AL-FF and Fit-FC methods are basically the same, with the difference that the optimal window is 9, and the AL-FF method is set adaptively, while the Fit-FC method is set manually and empirically to 9. In order to verify the performance and enhancement effect of the improved algorithm AL-FF, two sets of experiments were conducted to compare.

In Experiment 1, in order to explore the improved AL-FF algorithm, from the original Fit-FC’s three stages of Regression Model Fitting (RM), Spatial Filtering (SF), and Residual Compensation (RC) to two stages of Adaptive Sparse Regression Adapt Lasso-RM (AL-RM) and Residual Compensation Enhance-RC (ERC), the algorithm has become simpler and more computationally efficient. The enhancement of image detail capturing ability in the case of high The data used is Data 1: a three-band image of two pairs of Landsat-7 ETM+ and MODIS images [39] with green, red and NIR bands, the image is characterized by little spatial and spectral variations.

In Experiment 2, to explore the AL-FF captures complex nonlinear temporal phase changes. In addition, since the technical principles of various current fusion algorithms vary, each developed method is claimed by its original research to have unique advantages in terms of prediction accuracy, computational efficiency, or input data requirements. However, since these studies used different datasets in their method comparisons, it is difficult to determine which method is superior to all others. In general, the performance of different fusion methods mainly depends on the sensitivity to the spatial heterogeneity and spectral variations of the data used [42]. Therefore, numerous studies to assess the applicability of different data fusion methods for different application scenarios have conducted several cross-comparison studies based on time series data to explore the advantages and disadvantages of different methods [44,56,57]. Based on this, to judge the performance of the spatio-temporal fusion results of the AL-FF method with other methods, different scenarios in terms of spatial heterogeneity and spectral sensitivity are carried out to verify the algorithm’s applicability. The experiments of Experiment 2 include the comparison of image fusion results with significant heterogeneous spectral variations and the comparison of image results with both significant complex heterogeneous spatial variations and complex heterogeneous spectral variations. Data 2 was used: the Landsat 8 Operational Land Imager (OLI) surface reflectance product and the Moderate Resolution Imaging Spectroradiometer (MODIS) surface reflectance product were used as fine and coarse images, respectively. Their pre-processing process is delicate, the image quality is high, and the study area is two different types of regions, which satisfies us to perform a finer comparative analysis. For the experimental results, both quantitative and qualitative were used for judging. Quantitative way: the fusion accuracy of each experiment was quantitatively evaluated using root mean square error (RMSE), average absolute difference (AAD), structural similarity (SSIM) and correlation coefficient (CC). In general, the smaller the values of RMSE and AAD, and the larger the values of SSIM and r, the more accurate the prediction. The running time of each experiment was recorded to evaluate the effectiveness of the method; qualitative approach: intuitive visual evaluation was used to judge the enhancement effect.

## Results

### Comparison of Experiment 1 results

To verify the enhancement of the strategy proposed in this study applied to improve the Fit-FC algorithm at all stages. The results of Experiment 1 are analyzed and compared in terms of visual assessment, including the comparison between sparse regression model and regression model in terms of image detail capturing ability and image distortion, the superiority of adaptive window versus empirically set window, and the further enhancement of residual compensation based on the weight Function enhancement versus residuals directly introduced into the regression model of coarse spatial resolution image in terms of image spectral and detail capturing ability.

This section may be divided by subheadings. It should provide a concise and precise description of the experimental results, their interpretation, and the practical conclusions that can be drawn.

### Lasso Regression Model (LRM)

Fit-FC applies a linear regression model at coarse resolution to satisfactory resolution for prediction. Fine pixels that may have different temporal trends share the same coefficients, which leads to incorrect reflectance prediction and blurring effects. To solve this problem, in this study, the sparse feature of inter-image temporal phase variation is explored, and a light regression-based temporal phase variation estimation model is constructed to overcome the blurring of fused images caused by the inability of linear regression to capture complex nonlinear temporal phase variation.

**Fig 3:**
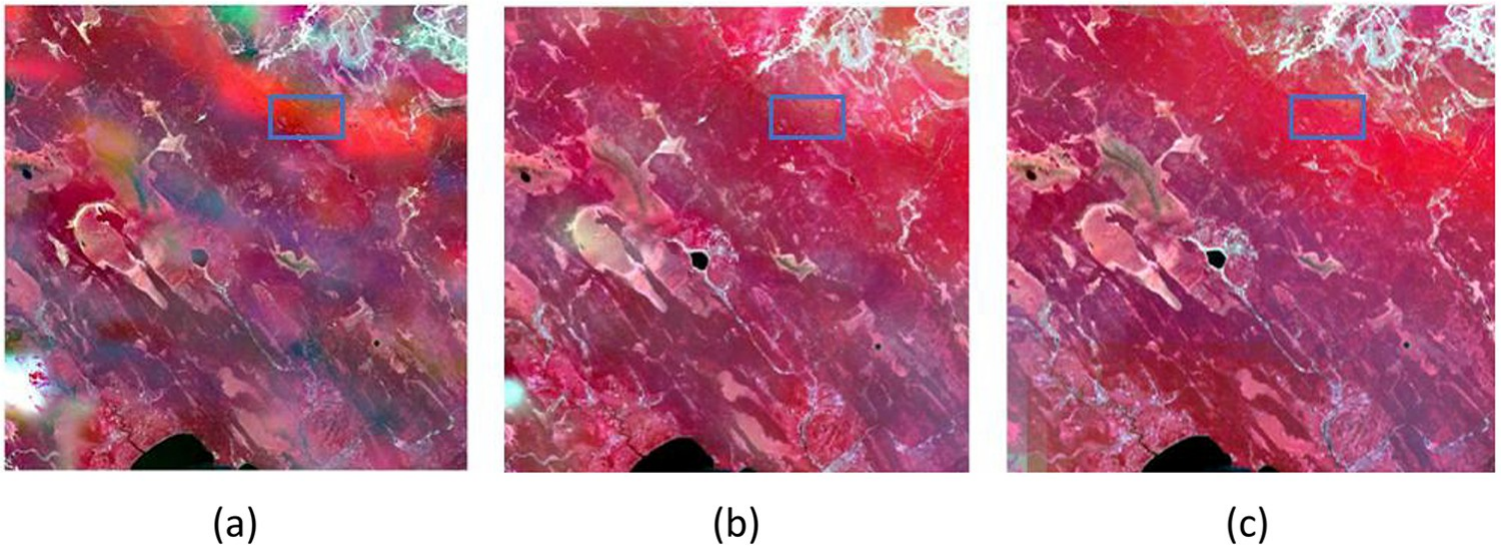
Comparison of the improved LRM and RM results. (a) is the fusion result of the RM stage of the Fit-FC algorithm. (b) is the fusion result of LRM of AL-Fit-FC algorithm.

In Fig 3. it can be seen that in the blue rectangle, the fusion result of the RM stage of the original Fit-FC algorithm (Fig 3A) is much weaker than the LRW fusion result of the first stage of the AL-Fit-FC algorithm (Fig 3B) in terms of the ability to capture the details of the image, which appears to be somewhat "fuzzy." Moreover, in the yellow rectangle area, the RM phase of the Fit-FC algorithm establishes the linear relationship between the coarse and fine remote sensing image pairs through the linear regression model, but it only captures some statistical features and cannot establish the complex nonlinear problem, which leads to the image distortion and artifacts [19]. The strategy of the AL-Fit-FC algorithm in the new RM phase is to explore the sparse characteristics of the temporal phase changes between images and to construct the sparsity characteristics based on the temporal phase changes of images and the sparsity characteristics of the temporal phase changes of images. Sparse characteristics and constructs a sparse regression-based temporal phase change estimation model, which overcomes the inability of linear regression to capture complex nonlinear temporal phase changes and thus leads to the blurring of fused images. In Fig 3(B), it can be seen that no artifacts are generated, which proves that our proposed strategy is successful. In addition to this, the AL-Fit-FC algorithm performs better than the original algorithm in terms of spectral features and image detail capabilities captured in both images as a whole.

#### Adaptive windows

**Fig 4:**
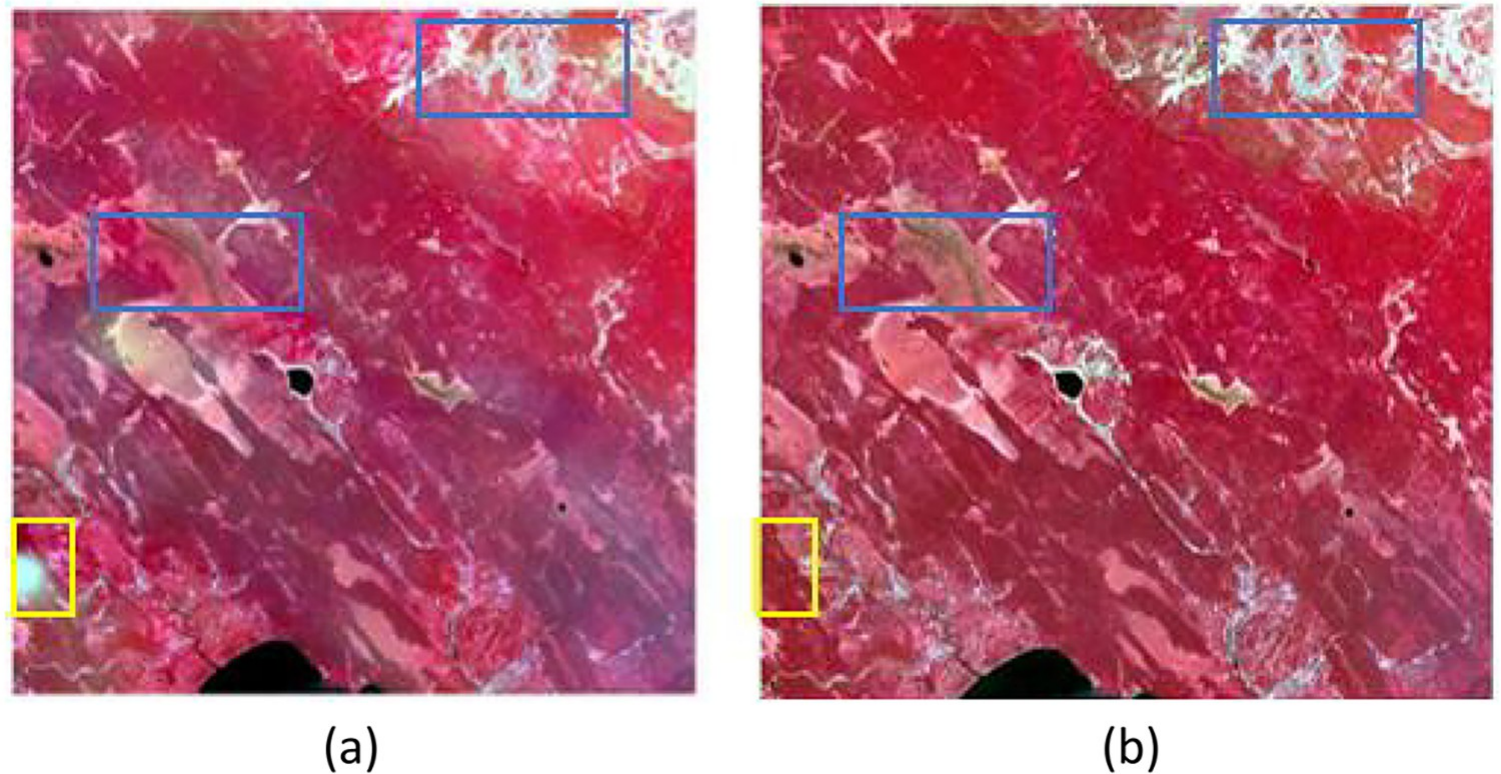
Result charts for different window sizes. (a), (b), and (c) represent the RM phase results of the original Fit-FC algorithm when the moving window size is 1, 9, and 12, respectively.

Based on the setting of the moving window size of the weighting Function, too large or too small will affect the accuracy of the final result, so according to the different input images, it is necessary to adjust the size of the window empirically constantly manually, and frequently set the parameters to adjust the size of the window [16].Fit-FC also inevitably needs to be adjusted every time the image is input to adjust the size of the moving window. The moving window is too large to lead to the fusion of the image being too smooth, and the moving window is too small, the fusion image will produce a particularly large number of artifacts, making the image distorted. If the moving window is too large, the image fusion will be too smooth, and if the moving window is too small, the fused image will produce a lot of artifacts, which makes the image distorted. In order to solve this problem, a strategy is proposed in this study: an adaptive window selection Function based on image gradient information and an adaptive weight setting Function are established, which overcomes the problem of manually setting the parameters on different datasets and improves the convenience of the algorithm and the robustness of the application on different datasets. Applying this strategy to the Fit-FC algorithm, the result of the AL-Fit-FC algorithm is shown in Fig 4, It can be clearly seen that the difference in the window size, the blue rectangular box inside the graphic details of the ability to capture different, in the window is smaller than 1 (Fig 4A), the image becomes particularly fuzzy, the distortion is more serious. Visually, most of the area is basically unable to determine the type of features; the window is larger for 12 (Fig 4C). We will see that the image is excessively smooth and accompanied by a small number of artifacts. Although the image can generally determine the type of features, it is not able to capture more detailed information in the image by constantly manually adjusting the size of the window and comparing the window with other windows of different sizes. We finally find the optimal window size of 9 (Fig 4B), which is the best window size. The optimal window size is 9 (Fig 4B), which is the best among all windows.

### Enhanced Residual Compensation (ERC)

Residual compensation based on weighting Functions is mainly designed to fully utilize the coarse spectral information to further enhance the image quality, but due to the simple use of interpolation for assigning the residuals, more detailed information is lost, and little spectral enhancement is achieved. In Fit-FC, the coarse residuals are resampled to a fine resolution using simple bicubic interpolation, redistributed through a similar weighting process in the SF step, and then returned to add SF predictions to preserve the spectral information. However, the results obtained in practice are very small, as shown in Fig 4, which shows the comparison between before and after residual compensation is performed. In this study, the residual compensation based on the weighting Function, which uses the spectral difference and temporal difference to construct the weighting Function, fully utilizes the spectral information, which not only leads to the improvement of the accuracy of the image but also leads to the further improvement of the detail capability.

**Fig 5:**
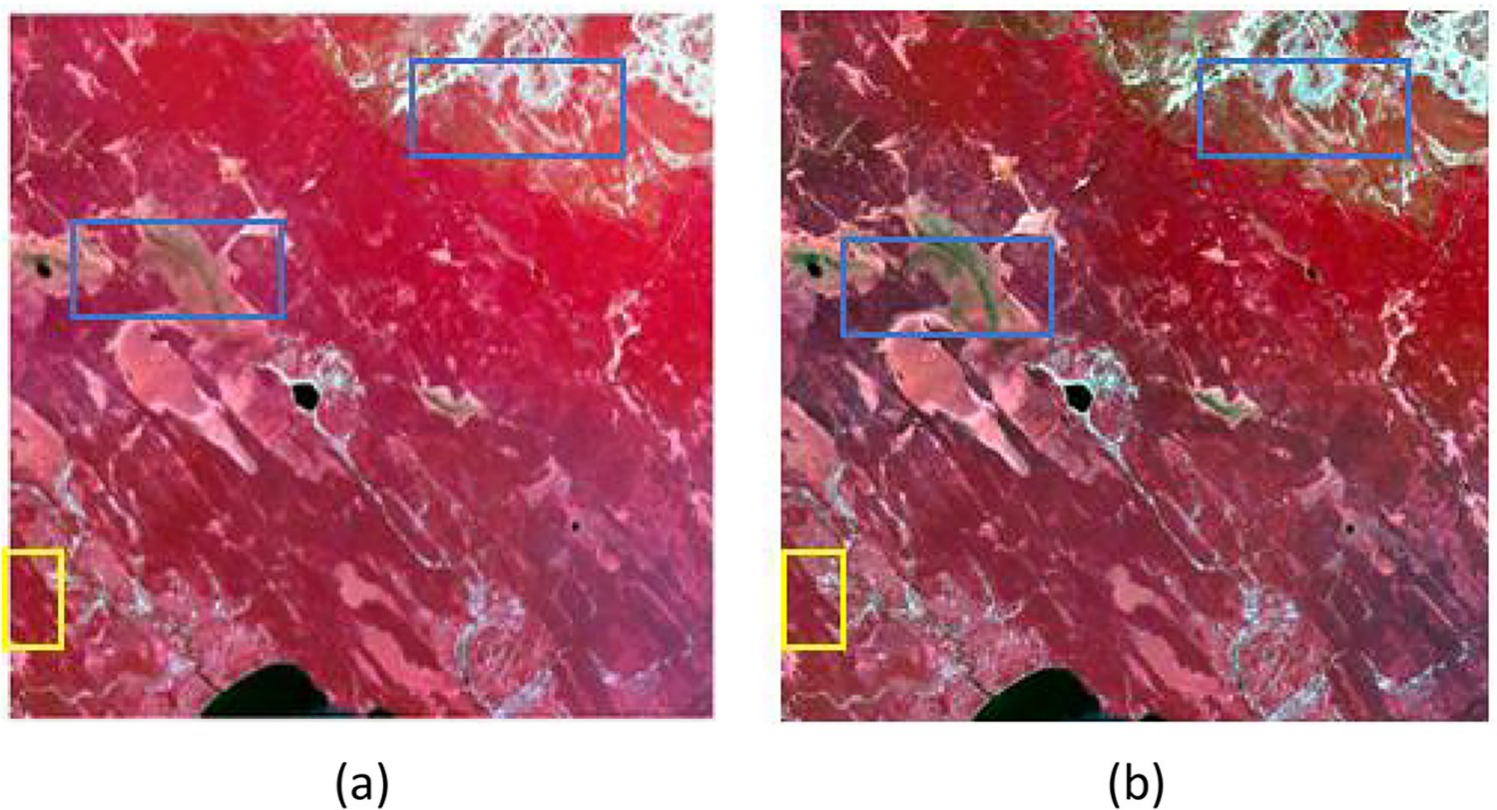
ERC of AL-FF and RC, RM results of Fit-FC. (a) represents the RW and RC phases of Fit-FC (b) represents the ERC phase of the AL-Fit-FC method.

In Fig 5, it can be seen that there are no artifacts inside the yellow rectangular box in both (a) and (b). The second stage of the Fit-FC method uses RW to remove artifacts, and the third stage uses RC for residual compensation. The AL-Fit-FC algorithm no longer produces artifacts in the first stage of the improved algorithm, and therefore, this stage mainly focuses on the enhancement of the image detail-capturing ability. Fig 3(B) compared with Fig 5(A), it can be seen that the improved algorithm not only does not produce artifacts in the first stage but also has surpassed the original algorithm’s three stages of image detail capturing ability in terms of detail capturing ability. The AL-Fit-FC algorithm, in the second stage of the ERC, adopts an improved strategy: the weight-based spatiotemporal fusion method searches for adjacent similar pixels and constructs a weight Function to predict the value of the center pixel. The center pixel value approach can introduce additional information about neighboring pixels and determine the contribution of neighboring pixels to the center pixel based on the weight Function, thus predicting the center pixel value more accurately, injecting fine spatial details, and using spectral differences, as well as temporal differences, to construct the weight Function, and no longer the original algorithm that only uses a simple bi-tertiary interpolation to resample the coarse residuals to a fine resolution. The detail capability of the image in the blue rectangular box in Fig 5B) is much better captured and is very close to the real features. In addition, it can be clearly seen that the ability to capture the vegetation information and the land cover change is greatly improved compared to both the three stages of the original Fit-FC method and the first stage of the AL-Fit-FC algorithm, which proves that the proposed strategy is feasible.

### Comparison of image fusion results of.AL-FF with other algorithms

To further evaluate the performance enhancement of the AL-FF method obtained by applying the strategy proposed in this study to the improved Fit-FC algorithm through spatial variations and spectral differences, experiments were conducted in two cases of complex heterogeneous spectral variations and complex heterogeneous spatial variations with significant variations, respectively.2 Hybrid-Based Approach Among all the spatial-temporal fusion algorithms, FSDAF is a well-known method for hybrid spatial-temporal data fusion, which takes into account abrupt land cover changes [25].FSDAF 2.0, an optimized version of FSDAF, improves the prediction of spatial details and land cover change regions by further evaluating the pixels that change after demixing [58]. In addition, RASDF introduces an adaptive local decomposition model that can retrieve strong temporal changes before filtering and assigning residuals [35].OL Fit-FC is a flexible object-level (OL) processing strategy proposed for the pixel-by-pixel fusion computation by weight Function-based methods, which leads to computational inefficiencies, and in a single-pair of experiments, OL Fit-FC’s detail-capturing ability performs the best. Therefore, we compared AL-FF with FSDAF 2.0, RASDF, Fit-FC, and OL-Fit-FC for qualitative evaluation of visual evaluation, as well as quantitative evaluation of the algorithms using spectral evaluation metrics: including Time (T), Root Mean Square Error (RMSE), Correlation Coefficient CC, Relative Global Dimensionless Error ERGAS, and Peak Signal to Noise Ratio PSNR, Average Absolute Error AAD. To make the experiments more accurate, we conducted 20 experiments at each site for each pair of input methods through simple permutations of the input images. The resulting large number of experimental results makes our evaluation of the fusion methods more comprehensive and reliable.

### Experiment 2 results visual assessment

#### Kherson

**Fig 6:**
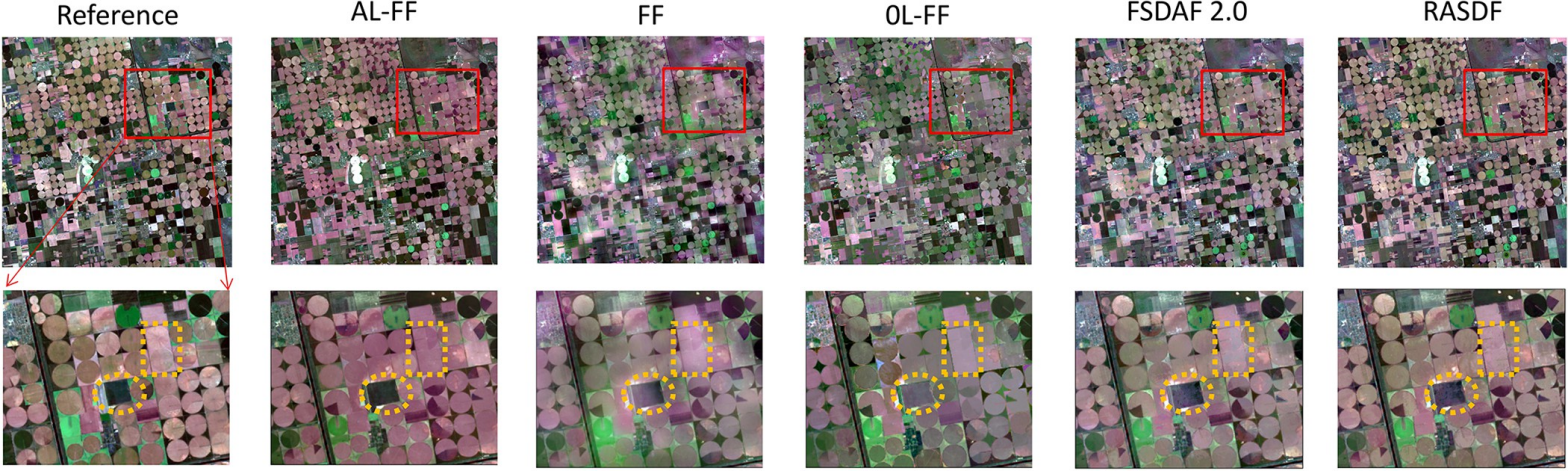
Results of the Kherson Area Visual Assessment.

The study site is located in the Kherson Oblast ("Kherson") irrigation area (33°31 22.85 E, 46°40 43.55 N) in southern Ukraine, where the shape of the agricultural land is rectangular or circular, with significant phenological variations between the images and slight differences in shapes, but with a wide variety of feature types, with different features all have significant spectral variations, thus having complex heterogeneous spectral variations. As shown in Fig 6, the AL-Fit-FC algorithm is closer to the actual features than other algorithms in terms of overall improvement of spectral features. However, its detail-capturing ability is not much different from other algorithms. The dotted line part retrieves significant climatic changes better than FSDAF 2.0, RASDF, Fit-FC, and OL-Fit-FC predictions, and the prediction is the clearest. For the region with more significant phenological changes, AL-Fit-FC performs better than the other algorithms, OL-Fit-FC is second best, and the other algorithms are much weaker. We also observe that inside the yellow dashed rectangle, it is obvious that AL-Fit-FC image quality is better; the brightness value, saturation, chromaticity, steepness, and size of the spectral texture features of the image are especially improved by AL-Fit-FC compared with other algorithms. It is the closest to the real image, while other algorithms have a large gap between the spectral color of most features and the real image after synthesis. However, in terms of detail-capturing ability, almost all algorithms failed to capture its small spatial changes.

#### PY

**Fig 7:**
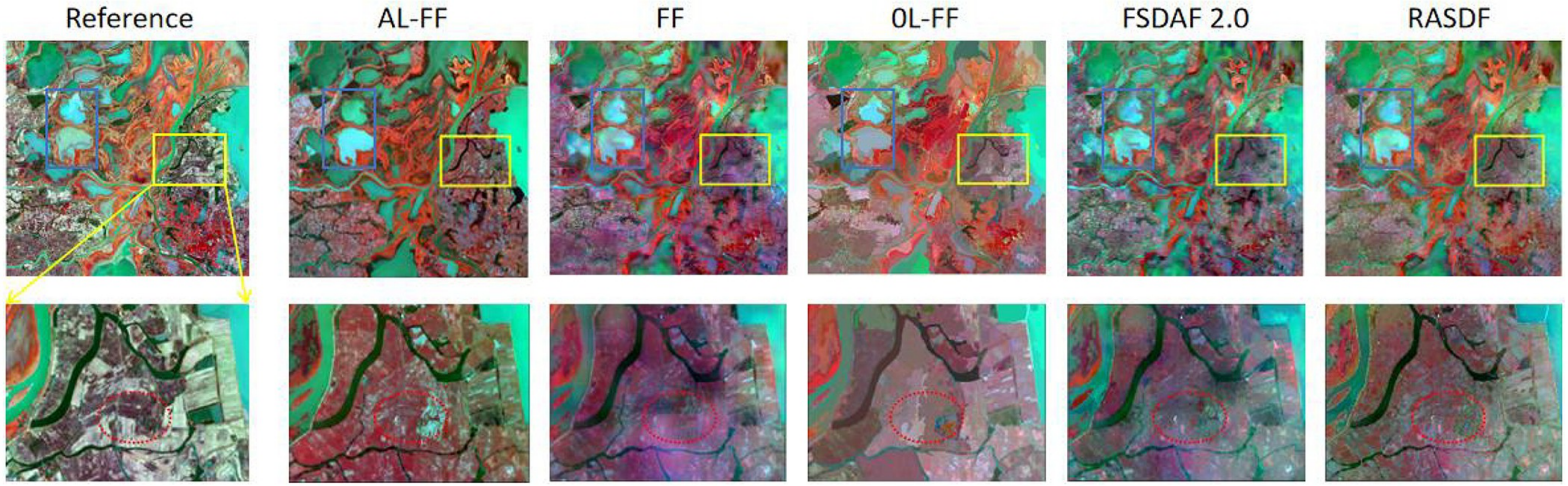
Results of the PY area visual assessment.

The study site is located in the southern part of Poyang Lake Wetland ("PY") (116°11 37.35 E, 28°57 57.39 N), Jiangxi Province, China, which has typical southern fragmented farmland, and has experienced significant shape changes due to the diversity of the PY watersheds, so that the different features not only undergo significant spatial variations but also accompanied by large spectral variations with complex heterogeneous spatial variations and complex heterogeneous spectral variations. As shown in Fig 7, the AL-Fit-FC algorithm shows better detail-capturing ability than other algorithms in the localized red dashed ellipse area. As a whole, the AL-Fit-FC algorithm also shows better detail-capturing ability than the other algorithms, which is especially obvious in regions with large land cover changes. Of all the algorithms, OL-Fit-FC is the least effective at capturing detail and is a bit blurry compared to the other algorithms. In the red dashed rectangle, only the AL-Fit-FC method captures the land cover changes, while the other methods do not; AL-Fit-FC retains more structural information and is very close to the real remote sensing image. Moreover, we can see that only AL-Fit-FC and RASDF algorithms can capture the narrow river in the red dashed ellipse area better, and AL-Fit-FC captures it more clearly and completely, while OL-Fit-FC, Fit-FC, FSDAF 2.0, and RASDF do not capture this tiny spatial detail clearly, which is enough to show that AL-Fit-FC compares with other algorithms to capture land cover changes, while all other methods do not capture it. Fit-FC will be more capable of capturing details than other algorithms. In addition to this, the locations with complex heterogeneous spatial variations in the dashed box also have complex heterogeneous spectral variations, but all the algorithms are not able to capture spectral features similar to the real features.

We found similar conclusions obtained at the same location with the same data source and algorithm parameters as Guo et al. [43], but with different conclusions in areas not shown in their paper. In Guo et al.’s conclusions, roughly in the blue rectangular dashed area in the figure, the mudflats were inundated by the lake during the fusion period, creating a clear change in spatial morphology, which was captured in better detail by the OL-Fit-FC algorithm than all other algorithms. We can also see in our figure that the OL-Fit-FC algorithm indeed outperforms all other algorithms in this aspect, with almost the same conclusion. However, in Guo et al.’s paper, the location of the lower-left region of our figure is not covered, which belongs to the region of urban buildings, and the detail capturing ability of OL-Fit-FC in this region is obviously the weakest, and the spectral features of OL-Fit-FC in this region are also the worst among all the algorithms, while our algorithm AL-Fit-FC shows the best performance in all the regions except for spatial variations of the water body.

### RMSE error maps

**Fig 8:**
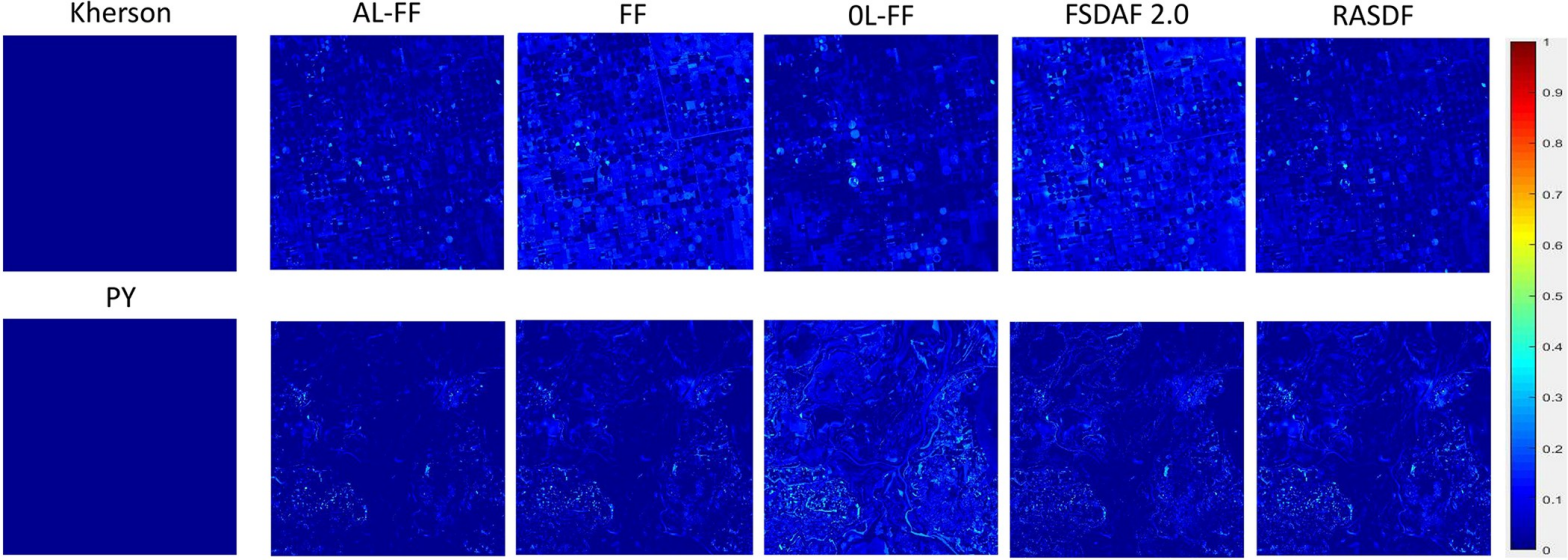
RMSE results.

To further visualize the fusion effect of each type of algorithm on various features in Kherson and PY areas as a whole. The global RMSE error map was used to carry out the representation of the fusion of various types of algorithms (Fig 8). The smaller the RMSE value, the higher the accuracy, which represents the higher correlation and structural similarity between the fused image and the real image, and the darker the blue color of the area in the map represents the smaller the value of the RMSE, and the better the fusion effect. Almost all the algorithms in the RMSE color in the water body part, water, and land boundaries are darker. Closer to 0, the effect is better. In addition to this, the AL-FF algorithm works better than the other algorithms in areas such as vegetation cover, dense urban buildings, mountains, etc. OL-Fit-FC has the worst effect in the HY region, with an overall too high RMSE value. However, the effect in the Kherson region, where the complex and heterogeneous spectra are large, is only second to the AL-FF algorithm.

The above experimental results and visual analysis show that the AL-Fit-FC method can retain structural information better than the Fit-FC, OL Fit-FC, FSDAF2.0, and RASDF fusion methods. In the "Kherson" region with little spatial variation and complex heterogeneous spectral variations, the spectral features of the fusion results of AL-Fit-FC compared with other algorithms are the closest to the real image, but almost all of the algorithms are unable to capture small spatial variations; in the "PY" region, which is characterized by complex heterogeneous spatial variations and complex heterogeneous hanging spectral variations, the fusion results of AL-Fit-FC compared with other algorithms are closer to the real image. In the "PY" area with both complex heterogeneous spatial variations and complex heterogeneous hanging spectral variations, the fusion results of AL-Fit-FC compared with other algorithms are better in capturing details, especially in the area with obvious land cover changes. However, the fusion results of almost all algorithms have significant spectral differences from the real images.

### Quantitative assessment of the results of Experiment 2

The quantitative evaluation is mainly done by correlation analysis using five evaluation metrics: root mean square error (RMSE), correlation coefficient CC, relative global dimensionless error ERGAS, peak signal-to-noise ratio PSNR, and mean absolute error AAD [19,51,52]. These metrics are used to quantitatively evaluate the similarity between the fused image and the actual image. The metrics of each algorithm in Experiment 2 are shown in Table 2, and the six band RMEs of each algorithm of Kherson and PY are shown in Tables 3 and 4, respectively.

**Table 2:** Results of different indicators for different fusion methods.

Sites	Indexes	FSDAF2.	RASDF	OL-FF	Fit -fc	AL-FF
Kherson	RMSE Time(s) ERGAS CC PSNR AAD	0.3738 561.143 0.9012 0.8227 18.6058 0.1054	0.2466 111.617 0.9467 0.8007 18.8524 0.0962	0.2300 23.169 0.8006 0.8272 20.4867 0.0629	0.2541 110.35 0.8922 0.8015 18.5851 0.1242	**0.2187** **1.0418** **0.7651** **0.9027** **20.8191** **0.0545**
PY	RMSE Time(s) ERGAS CC PSNR AAD	0.1196 686.469 1.6101 0.8336 16.1176 0.1354	0.1132 183.133 1.2807 0.8327 17.0869 0.1091	0.1189 35.98 1.3155 0.8008 16.9064 0.1134	0.0999 120.58 1.2409 0.8859 16.2649 0.0990	**0.0753** **1.3646** **1.0849** **0.9182** **17.4199** **0.0953**

**Table 3:** RMSE values of different algorithms for different bands in the "Kherson" region.

**Kherson**	**Methods**	**Blue**	**Gree**	**Red**	**NIR**	**SWIR**	**SWIR**
	Fit -fc	0.2542	0.2534	0.2522	0.2594	0.2544	0.2510
	**AL-Fit -fc**	**0.2144**	**0.2122**	**0.2056**	**0.2148**	**0.2235**	**0.2422**
	OL-Fit -fc	0.2250	0.2234	0.2153	0.2207	0.2357	0.2599
	RASDF	0.2508	0.2491	0.2458	0.2550	0.2429	0.2365
	FSDAF 2.0	0.3899	0.3860	0.3761	0.3739	0.3636	0.3538

**Table 4:** RMSE values of different algorithms for different bands in the "PY" region.

**PY**	**Methods**	**Blue**	**Gree**	**Red**	**NIR**	**SWIR**	**SWIR**
	Fit -fc	**0.0764**	0.1423	0.1304	0.0923	**0.0689**	0.0896
	**AL-Fit -fc**	0.1091	**0.0427**	0.0604	**0.0922**	0.0978	**0.0499**
	OL-Fit -fc	0.1665	0.1438	**0.0396**	0.0978	0.1412	0.1347
	RASDF	0.0993	0.0504	0.0802	0.1358	0.1656	0.1484
	FSDAF 2.0	0.0999	0.0994	0.1166	0.1252	0.1470	0.1297

The average RMSE, AAD, CC, PSNR, ERGAS, and total time Time(s) for the six bands in the dataset (blue, green, red, near-infrared, and short-wave infrared (SWIR1 and SWIR2)) are listed in Table 2. The best results for the AL-Fit-FC method compared to the original method and the other methods are highlighted in bold to emphasize the best results of the algorithm. The RMSE is more appropriate within 0–1, and the smaller, the better. In the Kherson region, the RMSE values are all very small, and AL-FF is the smallest at 0.218; in the PY region, the values are around 0.1, and AL-FF is the smallest at 0.075. the closer the CC value is to 1, the better the result is. All the algorithms are above 0.8, and AL-FF is above 0.9, which shows a very good performance. These indicate that the AL-FF method is effective in improving the correlation and structural similarity between the hybrid images and the corresponding real image observations.

It can be found that the effect of Fit-FC and FSDAF2.0 in PY with fragmented cropland and obvious morphological changes significantly improves the performance in the Kherson area with regular feature characteristics. RASDF and OL-Fit-FC have similar accuracy in the Kherson area and PY area. Still, OL-Fit-FC performs poorly in the PY area with complex heterogeneous spectral variations and complex heterogeneous spatial variations in comparison to Fit-FC and is worse than Fit-FC except for PSNR and Time(s). In addition, AL-Fit-FC obtained the best results in all the metrics of the experimental results in both cases of significant complex heterogeneous spatial variations and large complex heterogeneous spectral variations. Compared to the Fit-FC algorithm, the advantage is more obvious in the PY region (RMSE of 0.0753 and 0.0999, Time(s) of 3.3646 s and 120.58 s). It is worth noting that AL-Fit-FC has a 40–225 times improvement in computational speed compared to all algorithms and still has more than ten times improvement compared to OL-Fit-FC, which is the better computationally efficient algorithm among many current algorithms. In addition, the AL-Fit-FC algorithm has changed from the original Fit-FC’s three stages of regression model fitting (RM), spatial filtering (SF), and residual compensation (RC) to the improved sparse regression fitting Lasso-RM (LRM) and the improved use of spectral and temporal differences in constructing the weighting Function for residual compensation Enhance-RC (ERC). After the two stages, the computational process is simple, and the computational speed is improved by 40 to 55 times, which is a significant improvement.

Tables 3 and 4 show the corresponding RMSEs for the six bands (blue, green, red, near-infrared, and shortwave infrared (SWIR1 and SWIR2)) in the Kherson and PY datasets, respectively. The best results of the AL-Fit-FC method compared to the original method and other methods are highlighted in bold to emphasize the best results of the algorithm for each band. The fit-FC method performs best for blue and SWR1 bands in the PY region, OL- Fit-FC performs best for the red band, and the AL-FF method performs better for every band in the Kherson region, especially for blue, green, SWR1 band, OL- Fit-FC performs best for red band. Region in the blue, SWR1 bands, OL- Fit-FC performs best in the red bands, and the AL-FF method performs better in the Kherson region in every band, especially in predicting the blue, green, and red bands better than the NIR, SWR1, and SWR2 bands. These show that the AL-FF algorithm not only has the smallest RMSE overall but also remains the smallest in a single band and that the AL-FF method is the best compared to the other algorithms in terms of correlation and structural similarity between the fused images and the corresponding real image observations.

## Discussion

### Performance analysis

The AL-FF algorithm was able to completely remove the artifacts in the results of Experiment 1. This is because Fit-FC applies a coarse-resolution linear regression model to high-resolution images in a localized RM step, which allows fine pixels that may not share the same temporal trend to share the same change relationship for prediction. This inevitably introduces block artifacts, although SF attempts to mitigate it [19,59]. In the AL-FF method, on the other hand, this helps to preserve the spatial details well because this study explores the sparse nature of temporal phase changes between images and constructs a sparse regression-based temporal phase change estimation model, which overcomes the inability of linear regression to capture complex nonlinear temporal changes and thus blurring of the fused image, and captures the most essential features of the image, which is no longer a simple capture of statistical features.

The results of Experiment 2 show that the strategy proposed in this study can significantly improve the computational speed for two main reasons. On the one hand, there are two reasons for the computational inefficiency of most spatio-temporal data fusion methods: pixel-by-pixel computation and moving window strategy [28]. For weight Function-based methods, the weight of each acceptable pixel needs to be calculated from spatial, spectral, and temporal perspectives. Specifically, coarse and fine pixel modeling needs to be performed pixel by pixel. Also, many computations are realized within a moving window, e.g., STARFM-type methods typically use a moving window to search for similar pixels. The computational time requirement of these methods increases dramatically with the size of the moving window. The Fit-FC algorithm, on the other hand, performs linear modeling outside the moving window first, then removes artifacts by scanning the fuzzy pixels using the moving window, and finally, in order to make full use of the coarse-resolution image pairs, it performs residual compensation to improve its image quality. Therefore, compared with STARFM, ESTARFM, STI-FM, and other methods that directly use the moving window for pixel-by-pixel scanning, the computational speed is faster, and the fusion effect in terms of physical climate is better. In this study, the nonlinear time-phase problem is considered in the establishment of a linear relationship, so the artifacts are avoided in the results of Experiment 1, and thus, there is no need to remove the artifacts, which makes the computational efficiency higher as a matter of course. On the other hand, the AL-Fit-FC algorithm is able to avoid these problems completely by setting the adaptive window and finding the optimal moving window size quickly, which greatly saves time, improves efficiency, and avoids chance error. This makes it inevitable to play a great role in future studies of large-scale and long-time sequences.

RMSE is classical and precise in evaluating quantitative metrics and is capable of evaluating radiometric and structural errors [60]. In Table 2 of Experiment 2, the AL-FF method has higher data for all metrics than other advanced algorithms. But RMSE performs differently in different regions of the single band. The Kherson region in Table 3 is the best performance of the algorithm, and all the metrics are higher than other algorithms. However, in the PY region in Table 4, the performance in the blue, red, and short-wave infrared (SWIR1) bands is not the best, but the difference is not large, probably because surface reflectance is generally used in spatiotemporal fusion, where the amplitude of the red band is negatively correlated with the density of vegetation, and subject to the scale constraints, the typical fusion scenarios may include woodland, farmland, and grasslands, resulting in rich structure in the red band. The red spectrum varies greatly with space, leading to significant variations in the red band; the blue band is less intense, less detailed, and loses more detail in the case of large spatial variations; and the short-wave infrared (SWIR1) is most susceptible to the effects of time, which is spatially smooth, and loses a lot of detailed information in the case of complex spatial variations. Therefore, in contrast, the green band and other bands are less sensitive to spatial variations, so the green light and other bands continue to perform the best.

### Robustness

In general, large changes in space are accompanied by large changes in the spectrum, so it is difficult to carry out experimental investigations with only spatial changes and no spectral changes. In this study, we tried to investigate the case of only spatial changes without spectral changes through simulation experiments, but because it is not a real remote sensing image, we could not get a correct result, so we could not carry out the investigation of only spatial changes without spectral changes in Experiment 2. The result comparing with other algorithms, it can be seen that the spectral features of the AL-FF method are closest to the real image. In addition, the detail capability of the image in the blue rectangular box in Fig 6(B) of the results of Experiment 1 is captured better and the spectral features are very close to the real features.

In this study, in the Kherson area with complex heterogeneous spectral variations, the spectral features of the fusion results of AL-FF compared to other algorithms are closest to the real image. This is because the Fit-FC algorithm itself performs better than other algorithms when the physical changes are large [59], and the AL-FF method searches for neighboring similar pixels and constructs a weight function to predict the value of the center pixel by means of weight-based spatio-temporal fusion, which can introduce additional information about neighboring pixels and determine the contribution of the neighboring pixels to the center pixel based on the weight function, thus predicting the center pixel value with greater accuracy and injecting fine spatial details, and using a spectral fusion algorithm. accurate prediction of the center pixel value with fine spatial details injected, and uses spectral differences, as well as temporal differences, to construct the weight function, instead of the Fit-FC algorithm that only uses a simple bicubic interpolation to resample the coarse residuals to a fine resolution.

### Limitations of AL-FF

The AL-FF method is computationally efficient and capable of generating accurate surface reflectance fusion results, but still has some shortcomings. Firstly, our algorithm mainly focuses on the problem that the establishment of linear relationship cannot capture the complex nonlinear temporal phase and proposes a sparse regression-based solution strategy to capture more accurate feature information. However, the temporal changes of features are very complex, and the sparse regression-based model still has difficulty in capturing all the complex temporal changes, which leads to spectral distortion. On the one hand, the proposed method is still limited in its ability to capture spatial details due to the unestablished spatial fidelity constraint. On the other hand, our algorithm is more heavily cloud-affected by the images, and finding cloud-free images may be challenging, and thus conducting large-area, long-time series studies still faces the impact of cloud contamination.

In the future, we will explore deep learning-based spatio-temporal fusion methods to further enhance the final fusion results with a view to obtaining better prediction impacts. In addition, we will also consider the study of spatio-temporal fusion for cloud-covered scenes, which can be truly large-scale and long-time series.

## Conclusions

Remote sensing images with high spatial and temporal resolution are significant in applied research in environmental monitoring, agricultural management, urban planning, energy management, and transportation management. Spatio-temporal fusion techniques provide an effective method for realizing observations of dense time series. Among multiple spatiotemporal fusion methods, regression model fitting, spatial filtering, and residual compensation (Fit-FC) exhibit the best performance, with some tolerance to geometric alignment errors. In addition, the method still performs well when there are significant physical changes between fused images. However, this method makes it challenging to capture the complex nonlinear temporal phase change features between images, which leads to the blurring of the fused images. In addition, the need to empirically set the size of the moving window for different datasets makes it difficult to consider the distribution of image features, which weakens the ability to retain structural information and makes the method perform poorly in capturing image structure and texture. In addition, the inefficiency of spatio-temporal fusion is a common problem. To overcome these limitations, this study proposes a simple and fast adaptive sparse constrained Fit-Fc spatio-temporal fusion method for remote sensing images called Adapt Lasso-Fit-FC (AL-FF). The method describes the theoretical basis, implementation process, and performance of the fast adaptive sparse-constrained Fit-Fc remote sensing image spatio-temporal fusion method, which realizes simple, fast, efficient, and high-quality image fusion.

The AL-ff algorithm has the capability of simple, fast, efficient and high quality image fusion. In future research, the AL-FF algorithm is mainly used to solve the difficult problems in the application of high temporal and spatial resolution remote sensing images. For example, it can play an important role in long-term and large-scale refinement monitoring in water quality monitoring, crop growth monitoring and yield assessment, and wetland dynamics monitoring. In addition, We will explore the deep learning techniques for extracting nonlinear time-varying features in spatio-temporal fusion to better mine and retain the time-varying information in the images. On the other hand, we will also explore the multi-temporal spatial and temporal fusion techniques to fully utilize the complementary information between multi-temporal remote sensing images to better recover the time change information.

The main contributions of this study include:

- Aiming at the problems of insufficient detail capturing ability and low method efficiency of Fit-FC methods, a fast adaptive spatio-temporal spectral fusion method, called AL-FF, is proposed. Overcoming the problem of linear regression’s inability to capture complex nonlinear temporal phase variations and thus blurring fused images. In addition, to overcome the problem of empirical parameter settings on different datasets, the convenience of the algorithm and the robustness of its application on other datasets are improved.
- AL-FF firstly constructs a temporal phase change model based on sparse regression to capture the complex changing temporal phases in spatiotemporal fusion. After that, to improve the efficiency of the method, based on the gradient information and the variance information of the input images, an adaptive window size determination Function and a weight parameter determination Function are constructed, respectively, which prevents the method from determining the parameters manually and empirically when applying it to different data, and significantly improves the method’s efficiency.
- Compared with other state-of-the-art models, the improved AL-FF achieves better fusion fidelity under spatio-temporal fusion with complex heterogeneous spectral and simultaneous complex spectral and spatial variations.

## Supporting information

S1 Fig All data files are available from the figshare database. **(** https://figshare.com/articles/journal_contribution/Supporting_information_zip/25054640 **).**
(ZIP)
